# Prevalence of burnout among German general practitioners: Comparison of physicians working in solo and group practices

**DOI:** 10.1371/journal.pone.0211223

**Published:** 2019-02-06

**Authors:** Annegret Dreher, Mirjam Theune, Christine Kersting, Franziska Geiser, Birgitta Weltermann

**Affiliations:** 1 Institute of General Practice and Family Medicine, University of Bonn, Bonn, Germany; 2 Institute for General Medicine, University Hospital Essen, University of Duisburg-Essen, Essen, Germany; 3 Department of Psychosomatic Medicine and Psychotherapy, University Hospital Bonn, Bonn, Germany; Charles Sturt University, AUSTRALIA

## Abstract

**Background:**

Studies from general practitioner (GP) populations from various European countries show a high prevalence of burnout, yet data from Germany are scarce and there are no data comparing GPs from solo versus group practices.

**Methods:**

This cross-sectional survey addressed all GPs from a German network of family medicine practices comprising 185 practices. Participants were asked to fill in a self-administered questionnaire addressing socio-demographic and job-related characteristics. The German version of the Maslach Burnout Inventory was used to measure the dimensions emotional exhaustion (EE), depersonalization (DP), and personal accomplishment (PA). Each participant was categorized as having high EE, high DP and low PA following pre-defined cut-offs.

**Results:**

A total of 214 GPs from 129 practices participated: 65.9% male, 24.8% solo practice. Of all GPs, 34.1% (n = 73) scored high for EE, 29.0% (n = 62) high for DP, 21.5% (n = 46) low for PA and 7.5% (n = 16) for all three dimensions. A higher risk for EE was found among female physicians, those unsatisfied with their job, those using few stress-regulating measures regularly and those reporting bad work-life balance. Burnout prevalence was higher in GPs in group than in solo practices (37.9% vs. 28.8% had high EE, 33.1% vs. 18.9% had high DP and 22.8% vs. 18.9% had low PA). A significantly higher prevalence of burnout symptoms was found in group practice employees compared to group practice owners.

**Conclusion:**

Burnout prevalence was higher among physicians in group practices compared to solo practices. In group practices, employed, young, female and part-time working physicians showed a higher burnout risk.

## Introduction

Burnout was first described by Herbert Freudenberger in 1974 as consequence of overcommitment of workers in social professions resulting in tiredness, dullness and physical complaints [1]. Further research summarized the symptoms of burnout to the three dimensions emotional exhaustion (EE), feeling of depersonalization (DP) and low personal accomplishment (PA) [2]. Burnout was found to have severe health impacts on affected individuals, for instance sleep disturbance, feelings of depression and anxiety, and an increase in alcohol and drug misuse [3].

According to the 2013 health report of one of Germany’s largest sickness funds, burnout accounted for 10 days of sick leave per 100 insured people in 2012. The average duration of illness was 37.1 days [4]. Although the concept of burnout and its relationship to chronic stress, depression and other mental health disorders is still under debate, numerous studies describe high burnout prevalence in different professional groups, especially workers in the health care sector. Creedy et al. (2017) report a prevalence of work-related burnout of 43.8% in a sample of 1037 Australian midwives [5] and a meta-analysis by Gomez-Urquiza et al. (2017) found about one third of emergency nurses to be burnt out [6]. Physicians have also been found to be especially prone to job-related burnout: In a study from the United States (US), Shanafelt et al. (2015) reported the risk of burnout in physicians to be twice as high compared to the general population [7]. In another US study of 2012, general practitioners (GPs) were found to be the physician specialty with the highest reported burnout rates [8]. In 2008, the European General Practice Research Network found a burnout prevalence of 32% to 43% among GPs from 12 European countries [9]. This corresponded to a Canadian study with similar results [10]. According to another Canadian study, the financial burden caused by physicians suffering from burnout is estimated as $213 million over the next 26 years. This reflects the value of services lost to the healthcare system if the physician does not work. It includes $185 million attributable to early retirement and $28 million attributable to reduced working hours. [11]

Several systematic reviews have reported significant associations between burnout among medical staff and poor quality of healthcare including an increased number of medical errors [12–14]. Burnout was also shown to have a negative impact on job performance and was associated with hostile attitudes towards patients, difficulties relating with co-workers or increased intentions to quit the job [3,15–17]. Different stressors leading to burnout in physicians have been identified, such as lack of influence/autonomy, high workload and lack of time for private life activities [18,19]. Certain personality traits commonly found in physicians, such as high control demand, perfectionism, inflexibility or overcommitment, have been discussed to promote stress in physicians [20]. Furthermore, Frazer et al. (1994) identified various interpersonal factors as relevant stressors in medicine such as uncooperative physician peers, lack of staff and uncooperative patients [21].

In Germany, registered physicians with own practices provide the majority of outpatient care. In 2017, there were 55,032 GPs, most of them trained in either general medicine or internal medicine [22]. In solo practices, which accounted for over 75% of GP practices in 2016 [22], physicians are self-employed and are responsible for making decisions regarding working hours, choice of staff, and designing the work-place and work processes. A solo physician is responsible for his/her own documentation and billing. In group practices, two or more physicians sign an agreement on sharing rooms, equipment, staff and patients. Physicians are medically responsible for patients, but share the financial responsibility for the practice. A third type of collaboration is practice sharing. In this case, physicians share rooms, equipment and staff but not patients. Practices remain economically independent, which means that each physician takes care of his/her own patients and is responsible for his/her own documentation and billing. [23] Practice owners may employ additional physicians regardless of the type of practice they own. Employed physicians may work part-time. They have patient responsibilities but have less influence on the choice of staff, practice equipment or work processes.

Although solo practices remain the most common type of practice in many European countries (Belgium 75%, Netherlands 72%, Czech Republic 80%), there is a trend towards group practices [24,25]. Group practices appeal to physicians due to better control over working hours, shared resources, higher funds for investment, and greater opportunities for process standardization [24]. At the same time, new organizational and interpersonal challenges arise from this practice type, such as sharing of financial resources, room and equipment sharing, communication issues or disparities in patient treatment between physicians. A Danish study with 216 physicians reported higher burnout incidence among physicians from other-than-solo practices compared to solo practices (13.2% vs. 9.1% new burnout cases in seven years) [26]. In contrast, Soler et al. (2008) did not find significant differences between EE or PA prevalence according to practice type, while DP high burnout was more common in group than in solo practices [Odds ratio (OR) 1.34 (1.28–1.41)] [9].

Given the importance of burnout for health care systems and the lack of data on burnout in German primary care physicians, this study aims to describe the prevalence of burnout among German GPs, to identify job-related factors and to analyze differences between GPs in solo and group practices. Within group practices, we aimed to compare burnout prevalence between group practice employees and group practice owners.

## Methods

### Study population and study design

This study surveyed all GPs from the GP practice network of the Institute for General Medicine, University Hospital Essen, Germany, comprising 185 practices. These practices are located in urban and rural regions of North-Rhine-Westphalia (Western Germany), an average distance of 30 km from the institute.

Data collection was carried out from April through September 2014. Practices (n = 185) were invited by mail and contacted by phone for further recruitment communication. During a subsequent on-site visit, a staff member of the Institute for General Medicine distributed self-administered questionnaires to physicians and practice staff, which were collected on the next day in sealed envelopes for data privacy. In a few cases the filled-in questionnaires were sent back to the Institute via mail. Practices refusing to participate were asked to answer a short questionnaire on key practice characteristics and reasons for non-participation. All participants provided written informed consent. As an incentive, participating practices received a voucher of a department store chain. The voucher’s value was calculated by multiplying 5€ with the number of practice staff (physician and non-physician staff) and was given out regardless of how many individual staff members participated.

The study was approved by the Ethics Committee of the Medical Faculty of the University of Duisburg-Essen, Germany (reference number: 13-5536-BO, date of approval: 24/11/2014).

### Study instrument

The self-administered questionnaire consisted of a first section containing sociodemographic characteristics (e.g., physicians’ age, sex, number of children, family status, number of people living in one’s household) and job-related characteristics (e.g., working status, employment level, working hours, number of patients per three months, years since medical license, working years in this respective practice, work satisfaction, whether physicians would choose another job if they could decide again, and whether their expectations regarding their job were fulfilled). The type of practice (solo vs. group practice) was taken from the Institute for General Medicine’s GP practice register. A separate section of the questionnaire asked for physicians’ engagement (never, sometimes, regularly) in 20 different stress-regulating measures such as doing sports, playing an instrument or talking to friends.

Burnout was measured using the German version of the Maslach Burnout Inventory (MBI-D) [27] consisting of 21 items (9 items measuring EE, 5 items measuring DP and 7 items measuring PA). Each item´s frequency was rated on a 6-point-Likert scale ranging from 0 (never) to 5 (very often). For EE and DP higher values imply a higher risk, while for PA lower values indicate a higher risk of burnout.

### Statistical analysis

As no reference data for the MBI-D are published yet, cut-off values for the classification of “high” burnout were based on the European General Practice Research Network (EGPRN) study by Soler et al. (2008). The authors defined high EE as a sum score of ≥ 27 points, high DP as a sum score of ≥ 10 points and low PA as a sum score of ≤ 33 points [9]. Choosing the best cut-off to determine burnout when using the MBI has been a frequent subject of discussion. In a systematic review, Rotenstein et al. (2018) described 47 unique approaches to defining burnout in various studies using the MBI. The most frequent method (17.2% of the included studies) was the one chosen by the EGPRN [28]. Taking into account that the MBI-D consists of one item less compared to the international version and uses a 6-point Likert scale instead of a 7-point-Likert scale, the maximum EE, DP and PA item sums of the MBI-D are lower. Therefore, cut-off values were eventually set to ≥ 22 for high EE, ≥ 8 for high DP and ≤ 24 for low PA in this study.

Socio-demographic and job-related characteristics were displayed according to practice type and according to high EE, high DP and low PA. Continuous variables were reported as mean values with their respective standard deviations when normally distributed and reported as median values with interquartile range when not normally distributed. Group differences were calculated using Fisher’s exact test for categorical variables and Mann-Whitney U test for continuous variables with the significance level set to α = 0.05. All missing values were listed separately.

All GPs who provided complete data on the MBI-D were included in the analysis. Statistical analysis was performed using IBM SPSS Statistics for Windows, Version 22 (Armonk, NY: IBM Corp.).

## Results

### Response rate and Non-responder analysis

The response rate to our study was 74.1% including n = 137 practices with n = 226 GPs. Non-responder analysis on a practice level showed that there were only slight differences between participating and non-participating practices regarding key practice characteristics: Neither the type of practice (solo vs. group practice, p = 0.288) nor quarterly caseload (<1000, 1001–1250, 1251–1500, >1500 patients per 3 months, p = 0.066) influenced participation in the study. The mean number of practice assistants per practice did not differ significantly between participants (5.3 ± 4.0) and non-participants (4.6 ± 2.7) (p = 0.35), and neither did the mean number of physicians per practice (2.3 ± 1.5 and 2.4 ± 1.2, p = 0.769).

### Study population

Data of 214 GPs from 129 practices were eligible for further analysis. Socio-demographic and job-related characteristics of the study population are displayed in Table 1. Median participant age was 52.0 (Q1-Q3 46–59) years and 65.9% (n = 41) of the study population were male. 24.8% (n = 53) physicians worked in a solo practice and 73.8% (n = 158) physicians worked in a group practice. Information on the practice type was missing for three physicians (1.4%). Two physicians working in group practices lacked information on working status (practice owner vs. practice employee).

**Table 1:** Characteristics of the participating physicians (n = 214): total population and stratified by solo and group practice.

		Total study population (n = 214)		Solo practice (n = 53)		Group practice (n = 158)		Group practice owner (n = 132)		Group practice employee (n = 24)	
Age	Median (Q1-Q3) Missing	52.0 (46.0–59.0) 19		55.0 (48.0–63.0) 8		51.0 (46.0–57.0) 11		52.0 (48.0–58.0) 10		40.0 (37.0–49.0) 1	
People in one’s household	Median (Q1-Q3) Missing	3.0 (3.0–4.0) 0		3.0 (2.0–4.0) 0		3.0 (3.0–4.0) 0		3.0 (3.0–4.0) 0		3.0 (3.0–4.0) 0	
People in one’s household < 18	Median (Q1-Q3) Missing	2.0 (2.0–2.0) 14		2.0 (2.0–2.0) 4		2.0 (2.0–2.0) 9		2.0 (2.0–2.0) 6		2.0 (2.0–2.3) 2	
Years since medical license	Mean ± SD Missing	23.0 ± 9.2 5		24.9 ± 9.8 1		22.4 ± 9.0 3		24.1 ± 7.7 2		13.5 ± 9.6 0	
Years of working in this practice	Median (Q1-Q3) Missing	13.0 (7.0–21.8) 2		20.0 (11.0–26.5) 0		11.0 (6.0–20.0) 2		13.0 (8.0–21.0) 1		1.5 (1.0–5.8) 0	
Stress-regulating measures used regularly	Median (Q1-Q3) Missing	4.0 (2.0–6.0) 0		4.0 (3.0–6.0) 0		5.0 (2.0–6.0) 0		5.0 (2.0–6.0) 0		4.0 (3.0–6.0) 0	
		n	%	n	%	n	%	n	%	n	%
Sex	Male Female Missing	141 73 0	65.9 34.1 0	44 9 0	83.0 17.0 0	95 63 0	60.1 39.9 0	90 42 0	68.2 31.8 0	5 19 0	20.8 79.2 0
Family status	Married Divorced Single Missing	188 8 16 2	87.9 3.7 7.5 0.9	48 1 3 1	90.6 1.9 5.7 1.9	138 7 12 1	87.3 4.4 7.6 0.6	115 7 9 1	87.1 5.3 6.8 0.8	22 0 2 0	91.7 0 8.3 0
Caring for a needy family member	Yes No Missing	50 162 2	23.4 75.7 0.9	12 41 0	22.6 77.4 0	38 118 2	24.1 74.7 1.3	33 97 2	25.0 73.5 1.5	4 20 0	16.7 83.3 0
Living alone	Yes No Missing	9 205 0	4.2 95.8 0	4 49 0	7.5 92.5 0	4 154 0	2.5 97.5 0	4 128 0	3.0 97.0 0	0 24 0	0 100.0 0
Working status	Practice owner Practice employee Missing	185 27 2	86.4 12.6 0.9	50 3 0	94.3 5.7 0	132 24 2	83.5 15.2 1.3	132 0 0	100 0 0	0 24 0	0 100 0
Employment level	Full time Part time Missing	190 20 4	88.8 9.3 1.9	53 0 0	100.0 0 0	134 20 4	84.8 12.7 2.5	117 12 3	88.6 9.1 2.3	16 8 0	66.7 33.3 0
Working hours per week	≤ 39 hours 40–59 hours ≥ 60 hours Missing	52 116 45 1	24.3 54.2 21.0 0.5	9 26 18 0	17.0 49.1 34.0 0	43 88 26 1	27.2 55.7 16.5 0.6	30 76 26 0	22.7 57.6 19.7 0	12 12 0 0	50.0 50.0 0 0
Number of patients per 3 months	<1000 1000–1250 1251–1500 >1500 Missing	77 33 49 44 11	36.0 15.4 22.9 20.6 5.1	16 8 17 11 1	30.2 15.1 32.1 20.8 1.9	59 24 32 33 10	37.3 15.2 20.3 20.9 6.3	54 22 28 28 0	40.9 16.7 21.2 21.2 0	5 2 4 5 8	20.8 8.3 16.7 20.8 33.3
Would choose job again	Yes No Missing	198 13 3	92.5 6.1 1.4	49 3 1	92.5 5.7 1.9	147 9 2	93.0 5.7 1.3	124 7 1	93.9 5.3 0.8	22 2 0	91.7 8.3 0
Expectations regarding job fulfilled	Yes No Missing	146 64 4	68.2 29.9 1.9	38 14 1	71.7 26.4 1.9	108 47 3	68.4 29.7 1.9	91 40 1	68.9 30.3 0.8	16 7 1	66.7 29.2 4.2
Satisfied with current job	Yes No Missing	201 11 2	93.9 5.1 0.9	50 2 1	94.3 3.8 1.9	149 8 1	94.3 5.1 0.6	124 8 0	93.9 6.1 0	24 0 0	100.0 0 0
Reports good work-life balance	Yes No Missing	123 88 3	57.5 41.1 1.4	29 22 2	54.7 41.5 3.8	93 64 1	58.9 40.5 0.6	77 55 0	58.3 41.7 0	15 9 0	62.5 37.5 0

For a comparison of characteristics of physicians working in group and solo practices, see Table 1. Physicians in solo practices were more likely to be male (p = 0.002), older (p = 0.006), working full time (p = 0.003), having more weekly working hours (p = 0.025) and having spent more years in this practice (p<0.001) than physicians in group practices. Among GPs in group practices, employed physicians were younger (p<0.001), more likely to be female (p<0.001), working part time (p = 0.004), having less working hours per week (p = 0.03), and had spent less years in this practice (p<0.001) compared to group practice owners.

### Burnout according to the MBI

**Fig 1:**
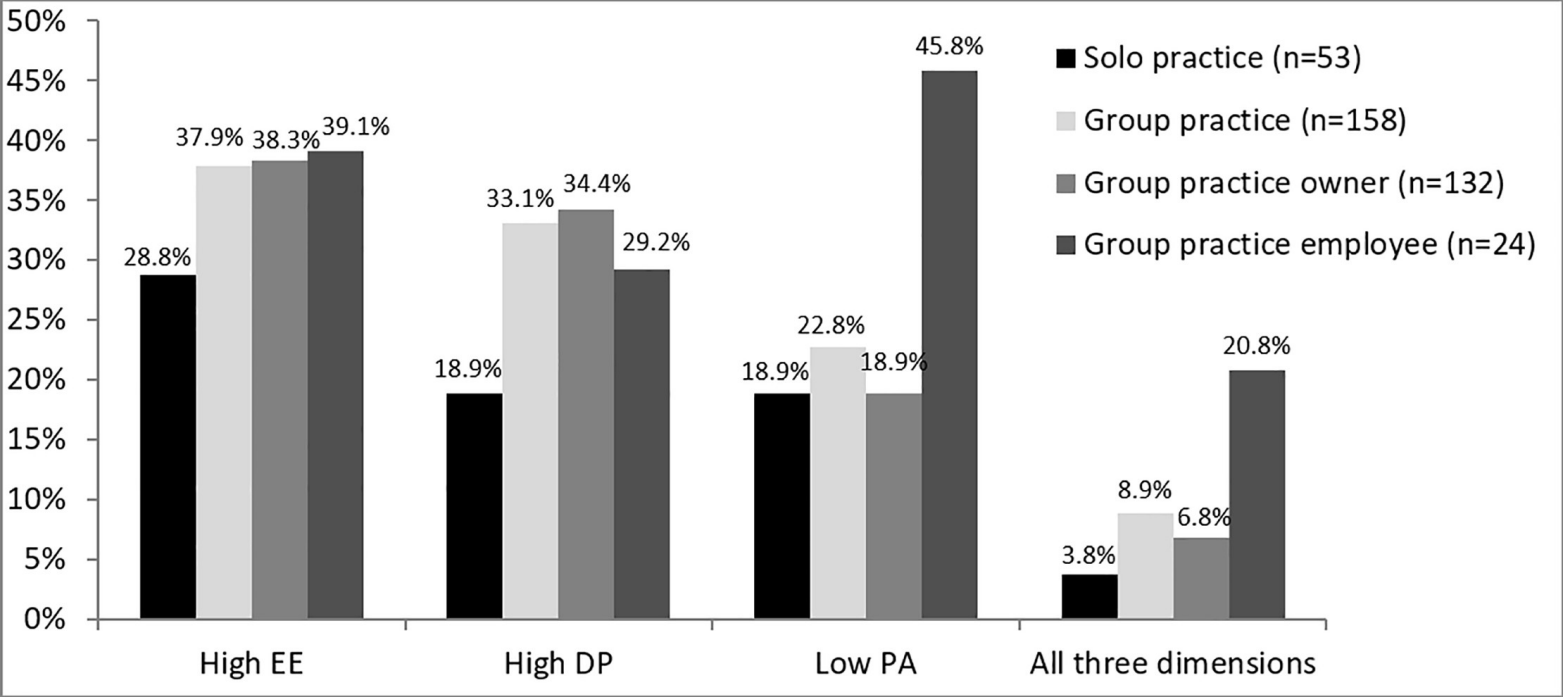
Burnout prevalence among general practitioners (n = 214) stratified by solo and group practices. Group practices (n = 158) were additionally stratified by working status (practice owner vs. practice employee); emotional exhaustion (EE), depersonalization (DP) and personal accomplishment (PA).

Median values and interquartile ranges of EE, DP and PA were 17 (11–25), 5 (3–8) and 28 (25–30), respectively. The overall prevalence of high EE (≥ 22 points) was 34.1% (n = 73), 29.0% (n = 62) had high DP (≥ 8 points), and 21.5% (n = 46) had low PA (≤ 24 points) (Fig 1). Considering burnout, 7.5% (n = 16) of physicians scored high in EE and DP and low in PA. Table 2 displays the prevalence of high EE, high DP and low PA according to socio-demographic and job-related characteristics.

**Table 2:** Socio-demographic and job-related characteristics of the participating physicians (n = 214) stratified by the three burnout dimensions high emotional exhaustion (EE), high depersonalization (DP) and low personal accomplishment (PA).

		**Total** **(n = 214)**	**High EE** **(n = 73)**		**High DP** **(n = 62)**		**Low PA** **(n = 46)**	
Age	Median (Q1-Q3) Missing	52.0 (46.0–59.0) 19	52.0 (46.0–58.0) 10		51.0 (46.0–56.5) 6		49.0 (44.0–55.0) 5	
People in one’s household	Median (Q1-Q3) Missing	3.0 (3.0–4.0) 0	3.0 (2.0–4.0) 0		3.0 (2.0–4.0) 0		3.0 (3.0–4.0) 0	
People in one’s household < 18	Median (Q1-Q3) Missing	2.0 (2.0–2.0) 14	2.0 (2.0–2.0) 6		2.0 (2.0–2.0) 5		2.0 (2.0–2.0) 4	
Years since medical license	Mean ± SD Missing	23.0 ± 9.2 5	23.08 (9.12) 1		21.43 (8.36) 1		20.02 (9.29) 1	
Years of working in this practice	Median (Q1-Q3) Missing	13.0 (7.0–21.8) 2	13.0 (8.0–21.0) 0		11.0 (6.0–18.0) 0		10.0 (6.0–18.0) 0	
Stress-regulating measures used regularly	Median (Q1-Q3) Missing	4.0 (2.0–6.0) 0	3.0 (1.0–5.0) 0		4.0 (2.0–6.0) 0		4.0 (1.0–5.0) 0	
			**n**	**%**	**n**	**%**	**n**	**%**
Sex	Male Female Missing	141 73 0	41 32 0	56.2 43.8 0	43 19 0	69.4 30.6 0	33 13 0	71.7 28.3 0
Family status	Married Divorced Solo Missing	188 8 16 2	66 2 4 1	90.4 2.7 5.5 1.4	56 2 4 0	90.3 3.2 6.5 0	40 2 4 0	87.0 4.3 8.7 0
Caring for a needy family member	Yes No Missing	50 162 2	16 56 1	21.9 76.7 1.4	12 49 1	19.4 79.0 1.6	16 29 1	34.8 63.0 2.2
Living alone	Yes No Missing	9 205 0	4 69 0	5.5 94.5 0	4 58 0	6.5 93.5 0	1 45 0	2.2 97.8 0
Working status	Practice owner Practice employee Missing	185 27 2	63 10 0	86.3 13.7 0	54 8 0	87.1 12.9 0	34 12 0	73.9 26.1 0
Employment Level	Full time Part time Missing	190 20 4	66 6 1	90.4 8.2 1.4	57 4 1	91.9 6.5 1.6	39 6 1	84.8 13.0 2.2
Working hours per week	≤39 hours 40–59 hours ≥60 hours Missing	52 116 45 1	13 43 17 0	17.8 58.9 23.3 0	21 32 9 0	33.9 51.6 14.5 0	13 27 6 0	28.3 58.7 13.0 0
Number of patients per 3 months	<1000 1000–1250 1251–1500 >1500 Missing	77 33 49 44 11	26 11 20 13 3	35.6 15.1 27.4 17.8 4.1	21 11 17 10 3	33.9 17.7 27.4 16.1 4.9	18 8 10 6 4	39.1 17.4 21.7 13.0 8.8
Would choose job again	Yes No Missing	198 13 3	64 7 2	87.7 9.6 2.7	54 8 0	87.1 12.9 0	41 5 0	89.1 10.9 0
Expectations regarding job fulfilled	Yes No Missing	146 64 4	44 28 1	60.3 38.4 1.4	30 31 1	48.4 50.0 1.6	24 21 1	52.2 45.7 2.2
Satisfied with current job	Yes No Missing	201 11 2	64 8 1	87.7 11.0 1.4	55 7 0	88.7 11.3 0	41 5 0	89.1 10.9 0
Reports good work-life balance	Yes No Missing	123 88 3	21 52 0	28.8 71.2 0	36 26 0	58.1 41.9 0	24 22 0	52.2 47.8 0

Especially female GPs (p = 0.031), GPs using less stress-regulating measures (p<0.001), those with less people living in their household (p = 0.007), GPs unsatisfied with their job (p = 0.004) and those reporting a bad work-life balance (p<0.001) scored high on EE. Significant associations with high DP were found for physicians not willing to choose this job again (p = 0.023), those whose expectations regarding job are not fulfilled (p<0.001) and those not satisfied with the current job (p = 0.017). Significant associations with low PA were found for caring for a needy family member (p = 0.047), being a practice employee (p = 0.005), expectations regarding job not fulfilled (p = 0.01), no satisfaction with current job (p = 0.042), less stress-regulating measures (p = 0.013) and less years spent in this practice (p = 0.015). Especially those participants who were unsatisfied with their job or would choose another job if they could decide again scored high in all three dimensions (Table 2).

Physicians working in group practices scored higher on all three burnout dimensions compared to physicians from solo practices (high EE 37.9% vs. 28.8%, high DP 33.1% vs. 18.9%, low PA 22.8% vs. 18.9%). Yet these results were not significant. High values for all three dimensions were found in 8.9% of group practices versus 3.8% of solo practices. Within group practices, employed physicians were more often affected by low PA (p = 0.005) and burnout when considering all three dimensions than group practice owners (20.8% vs. 6.8%) (p = 0.037) (Fig 1).

## Discussion

In agreement with data from other European countries, we report a high prevalence of burnout as measured on three dimensions among German GPs: one third of GPs were emotionally exhausted (34.1%), roughly one third highly depersonalized (29.0%) and about one in five doctors (21.5%) felt low personal accomplishment. As a new finding, we showed that burnout prevalence was higher among physicians working in group practices compared to those working in solo practices. Our detailed comparison of group practice owners and group practice employees identified employed GPs, who typically are part-time working, young females, as the highest burnout risk group. This finding is of importance for the physician workforce and warrants discussion.

### Prevalence of burnout in various GP populations

A number of studies [2,9,10,29–34] have used the MBI [35] to describe burnout in GPs. The burnout prevalence found in our study lays in the midst of other published burnout literature with some studies reporting even higher prevalence of burnout in their samples of GPs [9,10] and others reporting less [26,31,33,36]. We found female GPs to be more commonly emotionally exhausted than their male colleagues, which is in accordance with Deckard et al. (1994), but contrasts with Soler et al. (2008) and Marcelino et al. (2012) who found men to score higher on EE values [9,18,31]. Physicians engaging in less than five stress-regulating measures showed higher EE values than those engaging in more than five measures. The protective effect of recreation especially for EE has been previously reported by Wu et al. (2012) [37]. In line with Dusmesnil et al. (2009) [38] and Deckard et al. (1994) [18] we found GPs with a higher workload (>60h/week), in particular, to be emotionally exhausted. De Valk and Oostrom (2007) identified living in a big household as a protective factor against physician burnout, which agrees with our findings of physicians scoring higher on EE when living alone or with less people in their households [39].

We found burnout rates to be higher in group practices compared to solo practices. This is in accordance with Pedersen et al. (2013) who found similar but non-significant results in their sample of 216 Danish GPs [26]. Yet, this result might seem surprising given the observed lower workload of physicians in group practices and the higher number of reported stress-regulating measures. This finding might be explained by stressors that are more common in group practices than in solo practices. Practice culture (including practice dynamics and collegial conflict) has been reported as one of the key sources of stress by GPs [40] and might be of greater importance in group practices. Our findings might also be explained by socio-demographic differences of physicians according to practice type. Physicians in group practices in our study were more likely female, younger and had spent fewer years in their respective practice compared to solo practice physicians. Future research is needed to give insight on stressors particularly in group practices which might underlie the higher burnout prevalence in this practice type.

In group practices, employed physicians showed higher burnout rates compared to group practice owners, which may be explained by less influence on management issues such as room sharing or staff choice than practice owners. This is underlined by strikingly low PA values among employed physicians. Experiencing conflicting role demands and loyalties in the work setting has previously been reported as a predictor of low PA [37].

High burnout was found consistently in physicians reporting they would choose another job if they could decide again, in physicians whose expectations were not fulfilled and those who were not satisfied with their job. Intention to leave one’s job was previously reported as strong predictor for burnout risk, and this predicted burnout even better than low job satisfaction [15,41–43].

### Critical appraisal of the burnout concept and the MBI-D

Burnout has been a frequent subject of discussion in recent literature. Nevertheless, the concept is still vague, with several attempts at a definition (see e.g. [44]) and over 160 published single symptoms [45,46]. Particularly the distinction/overlap between burnout and depression remains a major point for discussion in the literature [47–49]. Furthermore the suitability of the 3-dimensional-model (EE, DP, PA) for burnout measurement remains controversial as well as the suitability of burnout’s job-related origin for distinction from other psychological disorders [47]. Up to now there is neither a universal definition for burnout nor a clear distinction from other, somewhat overlapping psychopathologies. The international classification of diseases (ICD-10) does not list burnout as a distinct disease but as a state of vital exhaustion (ICD-Code Z73.0) within the supplementary section “Problems related to life-management difficulty”. The German Association for Psychiatry, Psychotherapy and Psychosomatics recommends physicians to use this code for patients showing burnout symptoms without the presence of a defined ICD-10 coded disease [50].

When quantifying burnout prevalence, the MBI is the most widely used tool in the literature, measuring the three dimensions emotional exhaustion, depersonalization and personal accomplishment. Maslach et al. (2001) highlight the tool’s ability to distinguish job-related neurasthenia from other psychological diseases [2]. However, the MBI should not be used as a diagnostic tool as no universally valid cut-offs for distinguishing between different degrees of burnout exist and up to now only physicians can make a diagnosis [50]. Criticism of the tool was addressed by Koeske and Koeske (1989) who have questioned the structural validity of the MBI, criticizing the lack of rationale for its 3-dimensional-structure and the questionable attribution of single items to certain dimensions [51]. In our study we used the German version of the MBI which consists of only 21 items compared to the original version comprising 22 items, and uses a different scaling. This results in a reduction of maximum achievable points per burnout dimension and requires different cut-off values compared to the original tool. Furthermore, up to now there are no reference data from the German general population providing information on the three MBI dimensions, so comparison between burnout in German GPs and the German general population was not possible.

### Strengths and limitations

Our cross-sectional study provides data from a practice network of GPs that has been shown to be representative of German GP practices [52]. The high response rate of this study is explained by the close communication of the Institute with GPs by face-to-face distribution of questionnaires and by physicians being especially interested in stress and burnout. Our questionnaire addressed a broad variety of job-related characteristics and individual stress-regulating measures. Due to the cross-sectional design of the study only associations and no causality can be concluded. Limitations addressing the MBI are mentioned above.

## Conclusion and perspectives

Addressing high burnout prevalence in physicians, protective factors (e.g. living in a big household, engaging in stress-regulating measures) and prevention strategies have been discussed [39]. The Royal College of General Practitioners launched a campaign *(“put patients first; back general practice”)* with a handful of strategies on how to react to high physicians’ workload [53]. A randomized controlled trial by Ireland et al. (2017) found significant reductions of stress and burnout in physicians undergoing a mindfulness training intervention [54]. Our results underline that such strategies must address the subgroup of young, employed and female physicians in group practices, who were shown to be the group most susceptible to burnout.

For future research, a qualitative research design is needed to give more detailed insights about what really matters to group practice physicians. This methodological approach might be more suitable in the context of burnout research than statistical approaches [55]. In addition, prospective interventional studies are needed to evaluate the effectiveness of burnout reduction measures.
